# An exploration of how guideline developer capacity and guideline implementability influence implementation and adoption: study protocol

**DOI:** 10.1186/1748-5908-4-36

**Published:** 2009-07-02

**Authors:** Anna R Gagliardi, Melissa C Brouwers, Valerie A Palda, Louise Lemieux-Charles, Jeremy M Grimshaw

**Affiliations:** 1 Toronto General Research Institute, 200 Elizabeth Street, 13EN-235, Toronto, Ontario, M5G2C4, Canada; 2 McMaster University, 1280 Main Street West, Hamilton, Ontario, L8S4L8, Canada; 3 St Michael's Hospital, 30 Bond Street, Toronto, Ontario, M5B1W8, Canada; 4 University of Toronto, 155 College Street, Toronto, Ontario, M5T3M6, Canada; 5 Ottawa Health Research Institute, 725 Parkdale Avenue, Ottawa, Ontario, K1Y4E9, Canada

## Abstract

**Background:**

Practice guidelines can improve health care delivery and outcomes but several issues challenge guideline adoption, including their intrinsic attributes, and whether and how they are implemented. It appears that guideline format may influence accessibility and ease of use, which may overcome attitudinal barriers of guideline adoption, and appear to be important to all stakeholders. Guideline content may facilitate various forms of decision making about guideline adoption relevant to different stakeholders. Knowledge and attitudes about, and incentives and capacity for implementation on the part of guideline sponsors may influence whether and how they develop guidelines containing these features, and undertake implementation. Examination of these issues may yield opportunities to improve guideline adoption.

**Methods:**

The attributes hypothesized to facilitate adoption will be expanded by thematic analysis, and quantitative and qualitative summary of the content of international guidelines for two primary care (diabetes, hypertension) and institutional care (chronic ulcer, chronic heart failure) topics. Factors that influence whether and how guidelines are implemented will be explored by qualitative analysis of interviews with individuals affiliated with guideline sponsoring agencies.

**Discussion:**

Previous research examined guideline implementation by measuring rates of compliance with recommendations or associated outcomes, but this produced little insight on how the products themselves, or their implementation, could be improved. This research will establish a theoretical basis upon which to conduct experimental studies to compare the cost-effectiveness of interventions that enhance guideline development and implementation capacity. Such studies could first examine short-term outcomes predictive of guideline utilization, such as recall, attitude toward, confidence in, and adoption intention. If successful, then long-term objective outcomes reflecting the adoption of processes and associated patient care outcomes could be evaluated.

## Introduction

Research, practice, and policy in the health care sector focus on improving the organization, delivery, and outcomes of care, while optimizing efficiency. Critical to achieving these objectives is the need for compliance with best practice according to currently available knowledge generated through research. Knowledge syntheses such as practice guidelines provide the evidence base for health care decision making [1,2]. Their development, dissemination, and implementation are intended to improve quality of care. Unfortunately, their impact remains limited as there continue to be many documented circumstances where they have not been adopted into practice [3-6]. Several issues challenge guideline adoption, including their intrinsic attributes, and whether and how they are implemented.

### Guideline attributes

The Appraisal of Guidelines Research and Evaluation (AGREE) instrument assesses guidelines based on their scope and purpose, stakeholder involvement, rigour of development, clarity of presentation, editorial independence, and applicability [7]. The criteria for applicability specify that, to improve uptake, guidelines should include information about anticipated organizational barriers, costs associated with adoption, and measures for audit and monitoring. The Guideline Implementability Appraisal (GLIA) instrument also recommends that guidelines explicitly identify the anticipated impact of adoption on individuals and organizations, and include measures by which performance of the recommended medical interventions or services can be evaluated [8]. Both tools were proposed by guideline experts, and may not reflect the features important to target guideline users, including clinicians, managers, and policy makers.

Studies eliciting clinician views on guideline attributes that influence utilization are few. Interviews were conducted with 25 general practitioners in the United Kingdom to understand guideline qualities associated with adoption of recommendations for asthma, coronary heart disease, depression, epilepsy, and menorrhagia [9]. In addition to credibility of the source and content, they also desired information about the resources required to deliver recommended care, and recommendations formatted in step-wise fashion to highlight how and when to deliver care. During focus groups, target users of the American College of Occupational and Environmental Medicine practice guidelines stated that the guidelines were too complicated to use quickly, and suggested a variety of easier-to-read formats [10]. A single observational study examined the association between guideline attributes and use by general practitioners in the Netherlands [11]. Over a three-month period, 61 general practitioners documented the details of patient care visits during which one of ten national guidelines was relevant. Out of 12,880 decisions made by physicians, 61% complied with guidelines. Recommendations that had been categorized as evidence-based, provided clear and specific advice on actions, and that did not require a change in existing practice routines, including re-organization of staff, acquisition of extra resources, and learning of new knowledge or skills achieved higher compliance. Self-doubt and training needs were identified as the two issues most influencing adoption by primary care teams of the National Institute for Health and Clinical Excellence's Schizophrenia guideline [12]. An expert panel engaged to consider five guidelines for musculoskeletal disorders that had been judged by the AGREE instrument to have excellent technical quality found them to be only moderately acceptable, citing lack of relevance to usual practice [13]. Notably the applicability domain scored low for most of the musculoskeletal guidelines (range 0.17 to 0.76 out of 1.00). In Ontario, Canada a total of 488 clinicians were sent 1,494 new questionnaires regarding attitude to 34 clinical practice guidelines produced between 1999 and 2002 [14]. Endorsement of, and intent to use the guidelines were predicted by applicability, acceptability, and comparative value. Thus, in addition to the elements in the AGREE and GLIA tools, clinicians appear to also value ease of use, clarity of evidence, competency and training requirements, and identification of other practice changes required to accommodate the recommendations.

Fewer studies have investigated the guideline attributes considered important, or that lead to guideline utilization by managers and policy makers. A systematic review of 24 studies involving 2,041 interviews with health policy makers found that inclusion of summaries with policy recommendations was commonly suggested as a factor that could enhance guideline utilization [15]. A survey of 899 managers and policy decision makers from across Canada revealed that accessibility through the internet increased guideline utilization by all decision makers in government, regional health authorities, and hospitals, while adaptability influenced guideline utilization by hospital managers [16].

### Guideline implementation

Limited utilization of guidelines may depend on whether and how they are implemented. Recent syntheses of guideline implementation research found that there is considerable variation in the observed effects within and across interventions by condition and setting of care [17]. Outside of experimental research there are few evaluations of whether and how guidelines are actively implemented [18]. Those available suggest that the responsibility for guideline implementation is unclear, resources for implementation are lacking and, as a result, many guidelines are passively distributed. Interviews and focus groups with 47 government policy officers, agencies, practice guideline developers, and practitioners in Australia about the implementation of six practice guidelines revealed that no uniform strategy had been employed apart from mailing and posting on a web site [19]. Telephone interviews with health professionals in the United Kingdom revealed they experienced difficulty in acquiring resources to fund guideline implementation, often turning to 'soft' money from pharmaceutical companies for educational meetings, a traditional type of continuing education that is considered largely ineffective [17].

Lack of knowledge about implementation processes may also contribute to the reliance on passive distribution methods. Health professionals have acknowledged that they are unfamiliar with, or confused about the concept and practice of implementation [20]. Emergency medicine professionals from 16 countries highlighted their lack of skill in implementation [21]. Interviews with individuals from 33 international research funding agencies revealed a widespread need to increase our knowledge about, and the practice of implementation [22].

To date there has not been a systematic analysis of guideline features that may improve adoption, or the factors that influence whether and how guidelines are implemented by sponsoring organizations. Their examination may reveal opportunities to improve guideline adoption. The purpose of the proposed research is to: develop a conceptual framework of guideline attributes that could be used to characterize the ease with which they can be adopted; and explore how sponsoring organizations implement guidelines, describing factors that influence these processes.

### Theoretical framework

**Figure 1:**
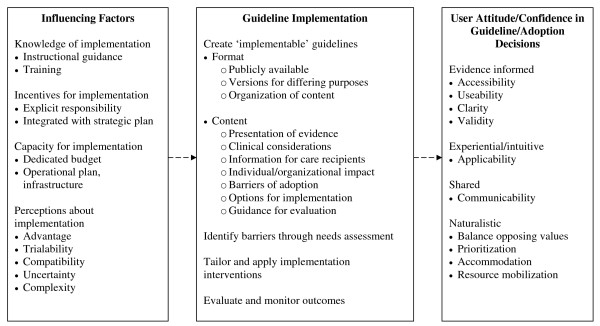
**Conceptual framework of factors influencing guideline development, implementation and adoption**.

To define the steps in guideline development and implementation, we draw upon the 'knowledge-to-action' (KTA) cycle, which involves synthesizing knowledge, adapting knowledge to the user context, assessing barriers of knowledge use, tailoring and applying implementation interventions, and evaluating outcomes (Figure 1) [20]. Knowledge and attitudes about, and incentives and capacity for implementation on the part of guideline developers may influence whether and how they undertake the KTA implementation processes [17-19]. Implementation can be considered a relatively new body of knowledge, so cognitive factors that may influence this practice will be examined, including perceived advantage (benefit over previous practice), trialability (control or autonomy over processes), compatibility (easy to undertake), uncertainty (facilitates organizational goals), and complexity (barriers) [23].

Clinicians, managers, and policy makers have suggested various guideline attributes that may enhance their 'implementability' [9-16]. Based on these studies, it appears that implementable features may improve attitude to the guidelines and confidence in decision making about adoption. Evidence is just one of several factors that inform clinical decision making [24]. Clinicians must often use experiential or shared decision making to consider what is best for and desired by those receiving care, but have expressed uncertainty about how to balance professional judgment with patient preferences, and the need for informational resources to support these processes. Clinician decisions about guideline adoption are also influenced by the availability and mobilization of organizational- or system-level resources, which are governed by the decisions of managers and policy makers who must consider not only evidence, but the benefits and risks associated with adoption, and the competing interests of multiple stakeholders, a process called naturalistic decision making [25]. Format elements of implementability are those that influence accessibility and ease of use, which may overcome attitudinal barriers of guideline adoption, and appear to be important to all stakeholders. Content elements of implementability are those that facilitate evidence-informed, experiential, shared and naturalistic decision making, stimulating confidence in whether and how to adopt guideline recommendations by different stakeholders.

## Methods

### Developing a conceptual framework of guideline implementability

The attributes hypothesized to facilitate adoption will be assessed and expanded by thematic analysis of the content of current guidelines. Published practice guidelines will be selected from among those reviewed (or the most recent version) by the Guidelines Advisory Committee http://www.gacguidelines.ca, a program in Ontario, Canada that identifies, appraises using the AGREE instrument, endorses and synthesizes guidelines, and were judged as high quality for two topics reflecting primary care (diabetes, hypertension) and two topics reflecting institutional care (chronic ulcer, chronic heart failure). Eligible guidelines include those that cover comprehensive management of these conditions, and are publicly available. Full versions of selected guidelines and adjunct products will be obtained. Data on presence of format and content features identified in the conceptual framework, or additional such features will be noted. Two individuals will independently extract data, then meet to compare findings and resolve differences. Extracted data will be tabulated. Most elements will be summarized quantitatively with mean, median, or frequency. Findings will be examined to discuss the number of guidelines addressing each element of implementability overall and by topic. Details of implementability content will be analyzed using Mays' narrative review method, based on verbatim reporting of information rather than statistical summary or conceptual analysis [26].

### Exploring factors influencing guideline implementation

Individuals affiliated with organizations that issue practice guidelines will be interviewed to explore the factors that influence guideline implementation. Standard methods of qualitative research will be used for sampling, data collection, and data analysis [27]. Individuals involved in sponsoring, developing, or implementing Canadian guidelines for four topics examined by content analysis will be identified on organizational web sites and through preliminary discussions with key contacts at those organizations (known sponsor approach). Ten consecutive individuals at each organization will be purposively recruited to represent different roles and perspectives, including sponsors, executives, managers, members of guideline development panels, and other individuals involved in coordinating guideline development or implementation, both internal and external to the involved programs, for a minimum total of 40 interviews. During interviews participants will be asked to recommend additional stakeholders that could provide relevant information (snowball sampling). Detailed information from representative, rather than a large number of cases is needed in qualitative research. Sampling is concurrent with data collection and analysis, and proceeds until no further unique themes emerge from successive interviews (grounded approach). If after 40 interviews new information continues to emerge, further interviews will be pursued. Data will be collected by conducting semi-structured telephone interviews with consenting participants. To enhance validity, a single investigator will conduct the interviews for internal consistency. They will be audio-recorded, then transcribed verbatim by an external professional. An interview guide will be pilot tested on one manager and clinician. Participants will be asked about their knowledge and perceptions of implementation; resources that were consulted to guide implementation decisions; their organization's incentives and capacity to implement guidelines; processes actually used for implementation; and suggestions for improving implementation capacity and processes. Unique themes will be identified in an inductive, iterative manner as previously described. Coded transcript text will be tabulated by theme and professional role.

## Discussion

Guideline implementability and implementation have not been systematically investigated to identify how they could be modified to improve guideline adoption. The development of a conceptual framework for implementability will be based on international guidelines for a variety of topics and therefore broadly applicable. Factors influencing the capacity for guideline implementation will be explored among a relatively small sample of participants in Ontario, Canada so those findings may not be relevant to guideline developers or sponsors in other settings with different types of health care systems, or where the organization of guideline development may differ from that in Ontario. Still, health systems worldwide experience non-compliance with guideline-recommended care, and seek novel insight into, and mechanisms for improving guideline utilization. The results of this study may provide a useful framework by which others can examine their capacity for guideline development and implementation.

With respect to policy and practice, this research may highlight that guideline development programs are not equipped to undertake implementation. Development of implementation capacity may be required to ensure that guideline sponsors and other groups seeking to improve quality of care have the required resources to achieve implementation. With respect to research, the findings will be used to refine the proposed conceptual framework, which could then inform ongoing studies. By identifying factors amenable to modification, for example, incorporation of actionable content within or as an adjunct product to guidelines, we establish a theoretical basis upon which to conduct experimental studies to compare the cost-effectiveness of such processes on the thoroughness of guideline implementation. Such studies could first examine short-term outcomes predictive of guideline utilization, such as recall, attitude toward, confidence in, and adoption intention [28]. If successful, then long-term objective outcomes reflecting the adoption of processes and associated patient care outcomes could be evaluated.

## Competing interests

The authors declare that they have no competing interests.

## Authors' contributions

ARG conceptualized and designed this study, prepared the proposal, and obtained funding. She will lead and coordinate data collection, analysis, interpretation, and report writing. She will be the primary investigator to independently review and extract data from interview transcripts and documents. MCB assisted with design of this study, and will oversee conduct of the document reviews, provide linkages with guideline development programs, and assist with interpretation and report writing. LLC assisted with design of this study, and will oversee conduct of the interviews, independently review interview transcripts, and assist with interpretation and report writing. VAP assisted with design of this study, and will independently review data extracted from guidelines, and assist with interpretation and report writing. JMG assisted with design of this study, and will independently serve as a third individual to resolve consensus differences, and assist with interpretation and report writing. All co-investigators contributed to the preparation of the funding proposal, and read and approved the final version of this manuscript.
